# Bipolar affective disorder

**DOI:** 10.1192/j.eurpsy.2021.44

**Published:** 2021-08-13

**Authors:** A. Young

**Affiliations:** Department Of Psychological Medicine, Centre For Affective Disorders, Institute of Psychiatry, Psychology and Neuroscience, King's College London, London, United Kingdom

## Abstract

**Abstract Body:**

**Abstract:** Biomarkers for diagnosis and treatment of Bipolar Disorder: hope or hype? Professor Allan Young, Centre for Affective Disorders, IoPPN, KCL London, SE5 8AF. allan.young@kcl.ac.uk The use of “biomarkers” (biological markers) in basic and clinical research as well as in clinical practice has become so commonplace in many areas of medicine that their presence as primary endpoints in clinical trials is now widely accepted. In clinical disciplines where specific biomarkers have been well characterized and repeatedly shown to correctly predict relevant clinical outcomes across a variety of treatments and populations, this use is entirely justified and appropriate. However, the validity of biomarkers in most psychiatric disorders continues to be evaluated. This lecture will review the current conceptual status of biomarkers as clinical and diagnostic tools for bipolar disorder and as surrogate endpoints in clinical research in bipolar disorder. The conceptual background in terms of current diagnostic categories and research domain criteria will be discussed and the various approaches with putative value (e.g., brain imaging, genetics, and neuroendocrinology) reviewed (1, 2). The lecture will end with a discussion of approaches to evaluating biomarkers of lithium response (3).

**Figure:**
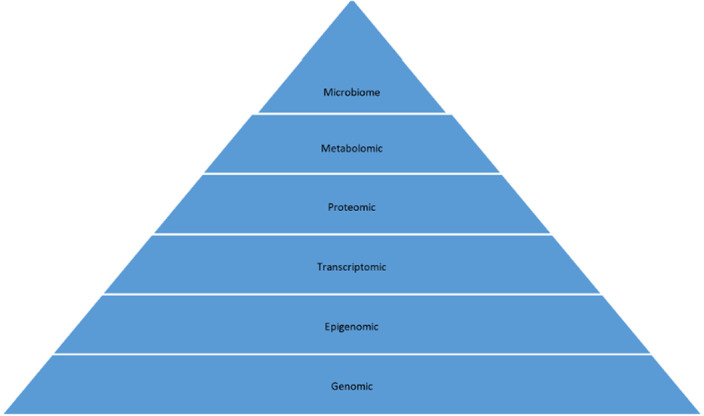

**References:**

Wise et al, Mol Psychiatry. 2016 May 24. doi: 10.1038/mp.2016.72. [Epub ahead of print]; Young AH. Harv Rev Psychiatry. 2014 Nov-Dec;22(6):331–3 Bellivier F, Young AH, et al, Bipolar Disord. 2020 Oct 23. doi: 10.1111/bdi.13023. Online ahead of print.

**Disclosure:**

Paid lectures and advisory boards for the following companies with drugs used in affective and related disorders: Astrazenaca, Eli Lilly, Lundbeck, Sunovion, Servier, Livanova, Janssen, Allegan, Bionomics, Sumitomo Dainippon Pharma, COMPASS Principal Inve

